# Neutrophil Gelatinase-Associated Lipocalin: A New Marker of Renal Function in C-Related End Stage Liver Disease

**DOI:** 10.1155/2015/815484

**Published:** 2015-06-28

**Authors:** Omkolsoum Mohamed Alhaddad, Ayman Alsebaey, Mohamed Omar Amer, Hala Hany El-Said, Tary Abdel Hamid Salman

**Affiliations:** ^1^Department of Hepatology, National Liver Institute, Menoufiya University, Shebeen El-Kom, Egypt; ^2^Department of Clinical Biochemistry, National Liver Institute, Menoufiya University, Shebeen El-Kom, Egypt

## Abstract

*Background/Aims*. Renal impairment is a common complication of cirrhosis. Serum creatinine is less sensitive in these patients. Measurement of the glomerular filtration rate (GFR) is the gold standard but time consuming. The aim is to validate plasma NGAL (pNGAL) and urinary NGAL (uNGAL) as markers of renal function in patients with HCV related cirrhosis. *Patient and Methods*. One hundred HCV related end stage liver cirrhosis patients were randomized into two groups: Group I (*n* = 35), patients with GFR < 60 mL/m measured by isotope scanning of the kidney (Renogram), and Group II (*n* = 65), patients with GFR ≥ 60 mL/m. The pNGAL and uNGAL were measured within 2 days of the Renogram. *Results*. Both groups were matched with age, sex, and Child Pugh score. There was statistically significant difference between both groups regarding serum creatinine (1.98 ± 1.04 versus 1.38 ± 0.88 mg/dL; *p* = 0.003) and pNGAL level (5.79 ± 2.06 versus 7.25 ± 3.30 ng/dL; *p* = 0.019). Both groups were comparable (*p* > 0.05) for the uNGAL (6.00 ± 0.78 versus 6.03 ± 0.96 ng/mL). Unlike uNGAL, the pNGAL positively correlated with total GFR by Renogram (*r* = 0.3; *p* = 0.001). With a cutoff ≥4 ng/mL, pNGAL had 94.3% sensitivity and 1.5% specificity and PPV = 34, NPV = 33.3, LR+ = −175.1, and LR− = −60.6. *Conclusion*. The pNGAL is a promising marker of the renal function in patients with cirrhosis.

## 1. Introduction

Liver cirrhosis is a dreadful complication of chronic HCV infection. It is complicated with the occurrence of esophageal varices, ascites, spontaneous bacterial peritonitis, dilutional hyponatremia, and hepatorenal syndrome (HRS) [1].

Renal impairment especially acute kidney injury (AKI) is commonly seen in patients with cirrhosis with increased risk of mortality [2]. It is usually precipitated by variceal bleeding, diuretics overuse, nephrotoxic drugs, for example, aminoglycosides, contrast media nephropathy, and abdominal paracentesis [2, 3].

Chronic kidney disease (CKD) is also seen in HCV related cirrhosis [4]. It is ascribed to the cryoglobulinemia and membranoproliferative glomerulonephritis [5]. Serum creatinine is the standard marker of the renal functions. It is synthetized in the liver as creatine, phosphorylated in the muscle to creatinine, to be removed by the kidney through filtration and active secretion. It is affected by age, gender, muscle mass, protein diet, and liver condition [6, 7].

Although it is commonly used to assess the renal function, it is a nonsensitive marker for renal dysfunction in patients with liver cirrhosis. This is due to decreased hepatic formation, malnutrition, loss of the muscle mass, and decreased secretion with spironolactone use. In addition, high bilirubin level impairs accurate measurement of serum creatinine by spectrophotometry (Jaffè method) [8, 9]. So, normal creatinine does not exclude renal impairment. Seeking for another surrogate is warranted.

Neutrophil gelatinase-associated lipocalin (NGAL) is 25 kDa protein of the lipocalin family [10]. It is synthesized in renal tubular, intestinal, hepatic, and pulmonary tissue. Its synthesis is upregulated markedly in tissue injury especially the kidney [11]. Circulating NGAL is filtered by the glomerulus to be reabsorbed in the proximal tubule. It is secreted in low concentrations by the thick ascending limb of the renal tubule [11, 12]. It can be measured in the serum and urine [13].

In proximal tubular injury, increased NGAL synthesis and decreased reabsorption occur causing increased urinary levels [12, 14]. In distal tubular injury there is increased distal renal tubular expression and synthesis of NGAL and NGAL secretion increases, increasing urinary levels as well [12, 15, 16].

In AKI, plasma NGAL levels rise, related to either concomitant hepatic, pulmonary, or intestinal tissue injury, coupled with decreased glomerular filtration of NGAL [11].

NGAL is a good predictor of AKI development, severity, and therapeutic monitoring [17] especially with postcardiac surgery, sepsis, renal replacement therapy, and rejection after kidney transplantation, reviewed in detail by Shemin and Dworkin and Haase et al. [11, 12].

This study aimed to validate plasma NGAL (pNGAL) and urinary NGAL (uNGAL) as markers of renal function in patients with HCV related cirrhosis.

## 2. Patients and Methods

This study was conducted in National Liver Institute hospitals, Menoufiya University, Egypt, after ethics committee approval and obtaining an informed consent from all the enrolled subjects.

One hundred HCV related end stage liver cirrhosis patients were included. All of them underwent isotope kidney scanning (Renogram) for the assessment of the glomerular filtration rate (GFR). They were divided into 2 groups by the GFR value: Group I (*n* = 35), patients with GFR < 60 mL/m, and Group II (*n* = 65), patients with GFR ≥ 60 mL/m.

The exclusion criteria were sepsis, GIT bleeding, concurrent medical disease such as long standing diabetes mellitus or hypertension, advanced intrinsic kidney disease as evidenced by small kidneys on ultrasound or morphological changes in static isotopic studies, solid organ transplantation, malignancy, and history of nephrotoxic drug administration.

All the patients underwent thorough history taking and physical examination. The following laboratory tests were done: liver function tests, blood urea, serum creatinine, serum sodium, serum potassium, CBC, and random blood sugar. Furthermore, abdominal ultrasonography with Doppler on the renal arteries for measuring the renal resistive index (RI) of both kidneys was done.

### 2.1. Sample Collection and Storage

For plasma and urinary NGAL measurement, blood and urine samples were collected within 2 days of the Renogram. Blood samples were drawn at the predetermined time points and processed within 2 hours after collection. Blood collected in serum separator tubes was allowed to clot for 15 to 20 minutes and then centrifuged for 12 minutes at 1000 g. Serum was collected and subsequently frozen at −80 Celsius until further analysis. Urine samples were centrifuged and the supernatant was collected and subsequently frozen at −80 Celsius until further analysis.

### 2.2. NGAL Analysis in Blood and Urine

All samples were analyzed in batches in a random fashion. The serum and urine NGAL level were performed using one of the commercially available assays (Wkea Med Supplies Corp.) that specifically detect human NGAL. The assay was performed as per the manufacturer's protocol. Briefly, 100 *μ*L of NGAL standards or diluted samples were applied onto the precoated microwells in duplicate. Microwells were then incubated for 1 h at room temperature and then washed with washing buffer. Finally, NGAL concentration was measured at 450 nm wavelength in each well. Urine creatinine was measured to standardize urinary NGAL for changes in urine concentration. Urinary NGAL excretion was presented as the amount of urinary NGAL in ng per mL urine as well as in ng per mg of urine creatinine to correct for differences in NGAL due to urine dilution. The laboratory investigators were blinded to the sample sources and clinical outcomes until the end of the study [18, 19].

### 2.3. Isotope Scanning of the Kidney

Tc-99m DMSA static renal scan; anterior, posterior, and posterior oblique views on the abdominopelvic region were acquired 3 hours after injection of 5 mCi of the tracer. Tc-99m DTPA dynamic renal scan; dynamic posterior views on the abdominopelvic region were acquired immediately after injection of 8 mCi of the tracer for 30 min, and I.V lasix was injected at the middle of the study. Then Renograms and renal function indices were generated. Delayed images were taken at 4 and 24 hours after injection.

### 2.4. Statistical Analysis

Data was statistically analyzed using IBM SPSS Statistics version 21 for Windows. Data are expressed as mean ± standard deviation. All *p* values are 2 tailed, with values <0.05 considered statistically significant. Comparisons between two groups were performed using Student's *t*-test for parametric data and Mann-Whitney test for nonparametric data. The linear relationship between two variables was analyzed by the correlation coefficient (*r*) (Pearson for parametric data and Spearman for nonparametric data). Univariate binary logistic regression was done for detecting the predictors of the GFR below 60 mL/m. The receiver operating characteristic (ROC) curve analysis was used for detection of the cutoff value of the plasma and urinary NGAL levels. Sensitivity, specificity, positive predictive value, negative predictive value, likelihood ratio positive, and likelihood ratio negative were used to express the cutoff. A value of 0.5–0.59 is of no useful performance for discrimination of the outcome under assessment.

## 3. Results

As shown in Table 1, both groups were matched for the age and gender. There was no statistically significant difference (*p* > 0.05) between Groups I and II regarding the MAP, CTP score, total bilirubin, serum albumin, AST, ALT, WBCs, platelets, and the INR.

However, a statistically significant difference was found between both groups regarding the MELD (23.71 ± 3.99 versus 20.09 ± 5.78; *p* = 0.002), MELD Na (28.40 ± 3.22 versus 25.69 ± 5.62; *p* = 0.025), and the hemoglobin level (9.60 ± 1.03 versus 10.54 ± 1.64 g/dL; *p* = 0.003).

Table 2 demonstrates the parameters of assessment of the renal function. There was a statistically significant difference between Groups I and II regarding the blood urea (101.94 ± 47.57 versus 78.23 ± 53.15 mg/dL; *p* = 0.03) and serum creatinine (1.98 ± 1.04 versus 1.38 ± 0.88 mg/dL; *p* = 0.003) unlike serum sodium and potassium. The renal resistive index measurement was not helpful as the values were comparable in both groups.

The pNGAL level was statistically significant between Groups I and II (5.79 ± 2.06 versus 7.25 ± 3.30 ng/mL; *p* = 0.019) contrary to uNGAL which was comparable between both groups without a significantly statistical difference (6.00 ± 0.78 versus 6.03 ± 0.96 ng/mL; *p* = 0.866).

The pNGAL level positively correlated with the GFR value measured by Renogram (*r* = 0.3; *p* = 0.001) unlike the uNGAL (*r* = 0.01; *p* = 0.848).

**Figure 1:**
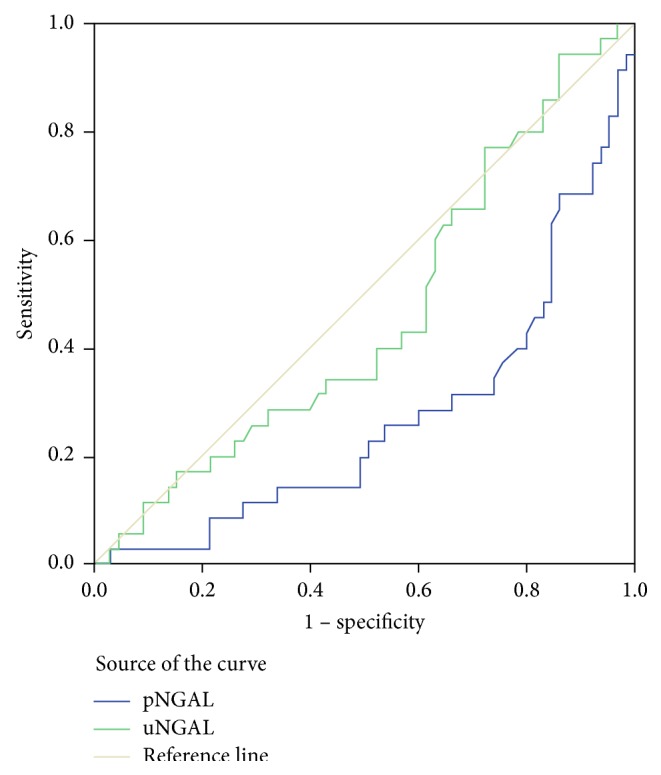
The receiver operating characteristic (ROC) curve analysis of pNGAL (*p* = 0.001) and uNGAL (*p* > 0.05).

The ROC curve analysis (Table 3 and Figure 1) revealed that the pNGAL was useful for detecting GFR below 60 mL/m (AUC 0.269; *p* = 0.001), unlike the uNGAL (AUC 0.459; *p* = 0.497).

The pNGAL is only a good positive test. A cutoff of 4 is associated with 94.3% sensitivity and 1.5% specificity and PPV = 34, NPV = 33.3, LR+ = −175.1, and LR− = −60.6. A cutoff of 5.3 is associated with 65.7% sensitivity and 13.8% specificity and PPV = 29.1, NPV = 42.9, LR+ = −5.11, and LR− = −4.7.

By using the logistic regression analysis the blood urea, serum creatinine, MELD, MELD Na, and pNGAL levels were predictors of the GFR below 60 mL/m.

By correlation analysis the total GFR correlated with serum creatinine (*r* = −5.25; *p* = 0.001), pNGAL (*r* = 0.329; *p* = 0.001), MELD (*r* = −0.525; *p* = 0.001), and MELD Na (*r* = −0.427; *p* = 0.001) unlike uNGAL (*r* = −0.182; *p* = 0.07). The serum creatinine did not correlate with either pNGAL (*r* = −0.120; *p* = 0.236) or uNGAL (*r* = 0.019; *p* = 0.848). The MELD score correlated with serum creatinine (*r* = 0.702; *p* = 0.001) in contrast to pNGAL (*r* = −0.06; *p* = 0.550). Furthermore the MELD Na score correlated with serum creatinine (*r* = 0.641; *p* = 0.001) in contrast to pNGAL (*r* = −0.037; *p* = 0.713).

## 4. Discussion

Renal impairment especially AKI is a common event in patients with cirrhosis [2, 4]. Despite the fact that serum creatinine is the standard marker of renal functions, it is a nonsensitive marker for renal dysfunction in patients with liver cirrhosis [8, 9]. Hence searching for new biomarker is highly needed. NGAL is a promising marker that may detect early AKI. Few studies investigated the usefulness of NGAL in patients with liver diseases. The earlier studies were mainly on liver transplantation (LT) recipients.

Niemann et al. [18] evaluated the AKI in 59 patients who underwent LT. In those patients with baseline serum creatinine <1.5 mg/dL preoperatively (*n* = 45), the baseline serum NGAL level was elevated. Measurement of intraoperative NGAL level was predictor of AKI development postoperatively. This was reinforced by Cheng et al. [20] who reported that measurement of pNGAL one hour after liver graft reperfusion was an early predictor of AKI.

Moreover Wagener et al. [21] found that urinary NGAL/urine creatinine ratio was able to predict postoperative AKI in LT patients. Furthermore Portal et al. [22] investigated cystatin C and NGAL as surrogate marker of renal function in patient who underwent LT. The pNGAL predicted the development of AKI within the first 48 hours after LT with high accuracy especially in those who required renal replacement therapy.

Fagundes et al. [23] were the first ones to study the utility of uNGAL among the patients with cirrhosis (*n* = 241). There were three groups with and without ascites and with renal impairment. The last group was subdivided into prerenal azotemia, CKD, HRS, and acute tubular necrosis (ATN). The uNGAL levels were higher in patients with renal impairment especially ATN compared to none (ATN > HRS1; ATN = HRS1 with infections; HRS type I > HRS type II, CKD, and prerenal azotemia). Furthermore it was higher in patients with urinary tract infection than in those without urinary tract infection.

Verna et al. [24] investigated uNGAL in predicting mortality and identification of HRS in patients with cirrhosis (*n* = 118). The levels of uNGAL in HRS were intermediate between prerenal azotemia and intrinsic kidney disease related AKI. Surprisingly uNGAL was predictor of the mortality and need for liver transplantation.

Barreto et al. [25] assessed the role of uNGAL in patients with cirrhosis with infection related renal impairment (*n* = 132). In fact, uNGAL was higher in patients with AKI than in those without, persistent AKI compared to transient. In addition, uNGAL was predictor of 3-month mortality.

Notably, most of the above studies did not measure the GFR by a standard method in contrast to our study. We use the isotope scanning of the kidney for accurate measurement of the GFR. Besides we used the renal resistive index by Doppler though it was insignificant statistically.

In our study only the pNGAL level was statistically different between the groups contrary to uNGAL. As to why the uNGAL did not increase, we have no explanation and moreover Sprenkle et al. [26] found that uNGAL did not appear to be a useful marker for detecting renal injury in healthy patients treated with partial nephrectomy.

Really in our study the serum creatinine level correlated positively with the MELD and MELD Na scores and negatively with the GFR so it still is useful marker of the kidney function. It did not correlate with both pNGAL and uNGAL. The pNGAL was only positively correlated with the GFR (*r* = 0.3; *p* = 0.001) despite being weak statistical correlation as *r* = 0.3.

The pNGAL level was a predictor of the GFR below 60 mL/m (odds = 0.691; *p* = 0.03). By using the ROC curve analysis the pNGAL level was a good positive test of high sensitivity and low specificity.

Despite these promising studies we should take into consideration also that there are some limitations with NGAL use. Most of the studies evaluated NGAL in homogeneous patients with single, acute, and easily identifiable nephrotoxic insults, such as cardiopulmonary bypass or intravenous contrast. NGAL appears to be less sensitive and specific in more heterogeneous cohorts with multifactorial causes for AKI [11]. The NGAL levels are elevated in CKD patients that are excluded in most studies despite being risk factor for AKI [27]. Uchino et al. [28] found that 30% of AKI patients were having CKD.

Malignancies and system infections, even simplest infection like UTI, are associated with elevated NGAL levels [11, 29]. NGAL may be more accurate in children when compared to adults who make up the vast majority of patients with AKI [11]. Finally the method of measurement may affect the results [11, 12].

In conclusion the pNGAL is a promising marker of the renal function in patients with liver cirrhosis.

## Figures and Tables

**Table 1:** Comparison of the baseline data in both groups.

	Group I	Group II	*p*
	GFR < 60 mL/m	GFR ≥ 60 mL/m	
	*N* = 35	*N* = 65	
	M ± SD	M ± SD	
Age (years)	51.17 ± 7.61	47.80 ± 8.90	0.06
Gender			
Female (*N*)	8	9	0.26
Male (*N*)	27	56	
MAP (mmHg)	80.86 ± 6.48	80.46 ± 6.87	0.780
CTP score	10.14 ± 1.48	10.08 ± 1.46	0.831
MELD^§^	23.71 ± 3.99	20.09 ± 5.78	0.002^*∗*^
MELD Na^§^	28.40 ± 3.22	25.69 ± 5.62	0.025^*∗*^
Total bilirubin (mg/dL)^§^	5.45 ± 5.64	4.16 ± 3.20	0.450
Albumin (g/dL)	2.48 ± 0.50	2.44 ± 0.45	0.733
AST (u/L)	74.11 ± 43.30	75.67 ± 43.51	0.865
ALT (u/L)	49.83 ± 26.90	52.62 ± 30.80	0.653
Hemoglobin (g/dL)^§^	9.60 ± 1.03	10.54 ± 1.64	0.003^*∗*^
WBCs ×10^3^/*μ*L	5.60 ± 3.19	5.74 ± 3.22	0.831
Platelets ×10^3^/*μ*L	103.57 ± 26.29	93.12 ± 51.53	0.265
INR	1.77 ± 0.30	1.69 ± 0.27	0.195

^§^Mann-Whitney *U* test, GFR: glomerular filtration rate, MAP: mean arterial pressure, CTP: Child Pugh score, and MELD: model of end stage liver disease; ^*∗*^ *p* < 0.05.

**Table 2:** Comparison of the renal function parameters in both groups.

	Group I	Group II	*p*
	GFR < 60 mL/m	GFR ≥ 60 mL/m	
	*N* = 35	*N* = 65	
	M ± SD	M ± SD	
Urea (mg/dL)	101.94 ± 47.57	78.23 ± 53.15	0.03^*∗*^
Creatinine (mg/dL)	1.98 ± 1.04	1.38 ± 0.88	0.003^*∗*^
Serum sodium (mmol/L)	127.46 ± 4.56	124.92 ± 16.50	0.376
Serum potassium (mmol/L)	4.31 ± 0.75	6.11 ± 14.99	0.482
Right kidney resistive index^§^	0.68 ± 0.06	0.66 ± 0.05	0.096
Left kidney resistive index	0.66 ± 0.04	0.66 ± 0.05	0.677
pNGAL (ng/mL)	5.79 ± 2.06	7.25 ± 3.30	0.019^*∗*^
uNGAL (ng/mL)	6.00 ± 0.78	6.03 ± 0.96	0.866

^§^Mann-Whitney *U* test; ^*∗*^ *p* < 0.05.

**(a):** 

	Area under the curve		Asymptotic 95% confidence interval	
	Area	*p*	Lower bound	Upper bound
pNGAL	0.269	0.001^*∗*^	0.164	0.373
uNGAL	0.459	0.497	0.341	0.577

**(b):** 

pNGAL cutoff	Sensitivity	Specificity	PPV	NPV	LR+	LR−
4 ng/mL	94.3%	1.5%	34	33.3	−175.1	−60.6
5.3 g/mL	65.7%	13.8%	29.1	42.9	−5.11	−4.7

PPV: positive predictive value, NPP: negative predictive value, and LR: likelihood ratio; ^*∗*^ *p* < 0.05.

## Abbreviations

AKI: Acute kidney injury
CKD: Chronic kidney disease
CTP: Child Pugh score
GFR: Glomerular filtration rate
HRS: Hepatorenal syndrome
MAP: Mean arterial pressure
MELD: Model of end stage liver disease
NGAL: Neutrophil gelatinase-associated lipocalin
pNGAL: Plasma NGAL
RI: Renal resistive index
uNGAL: Urinary NGAL.
